# Comparison of Pretreatment Strategies for cT2cN0 Staged Adenocarcinoma of the Esophagus and the Gastroesophageal Junction: A European High-Volume Center Cohort Analysis

**DOI:** 10.1245/s10434-025-18311-8

**Published:** 2025-10-13

**Authors:** Naita M. Wirsik, Thomas Schmidt, Cezanne D. Kooij, Niall Dempster, Nerma Crnovrsanin, Noel E. Donlon, Eren Uzun, Kunal Bhanot, Henrik Nienhüser, Daniela Polette, Kammy Kewani, Peter Grimminger, Daniel Reim, Florian Seyfried, Hans F. Fuchs, Suzanne S. Gisbertz, Christoph-Thomas Germer, Jelle P. Ruurda, Fredrik Klevebro, Wolfgang Schröder, Magnus Nilsson, John V. Reynolds, Mark I. Van Berge Henegouwen, Sheraz Markar, Richard Van Hillegersberg, Christiane J. Bruns

**Affiliations:** 1 Department of General, Visceral, Thoracic and Transplant Surgery, University Hospital of Cologne, Cologne, Germany; 2 Department of Surgery, University Medical Center Utrecht, Utrecht University, Utrecht, The Netherlands; 3 Nuffield Department of Surgical Sciences, University of Oxford, Oxford, UK; 4 Department of General, Visceral, and Transplant Surgery, University Hospital of Heidelberg, Heidelberg, Germany; 5 Department of Surgery, School of Medicine, Trinity College Dublin, Dublin, Ireland; 6 Department of General, Visceral and Transplant Surgery, University Medical Center of the Johannes Gutenberg University, Mainz, Germany; 7 Department of Upper Gastrointestinal Diseases, Karolinska University Hospital, Stockholm, Sweden; 8 Department of Surgery, Amsterdam UMC Location University of Amsterdam, Amsterdam, The Netherlands; 9 School of Medicine and Health, Department of Surgery, Technical University of Munich, Munich, Germany; 10 Department of General, Visceral, Transplantation, Vascular and Pediatric Surgery, University Hospital Wuerzburg, Wuerzburg, Bavaria Germany; 11 Trinity St James’ Cancer Institute, St James’s Hospital Dublin, Dublin, Ireland; 12 Division of Surgery and Oncology, CLINTEC, Karolinska Institute, Stockholm, Sweden

**Keywords:** cT2, Adenocarcinoma, Pretreatment

## Abstract

**Background:**

After the ESOPEC trial showed a survival benefit for fluorouracil, leucovorin, oxaliplatin, and docetaxel (FLOT)-treated adenocarcinomas of the esophagus (EAC) and the gastroesophageal junction (GEJ) compared with Chemoradiotherapy for Oesophageal Cancer Followed by Surgery Study (CROSS), a European, high-volume center study for stage cT2cN0 EAC and GEJ was undertaken, as it has been published that a third of these patients are understaged and could benefit from a multimodal approach

**Patients and Methods:**

Retrospective analysis of prospective databases from ten high-volume European centers was performed. Inclusion criteria were GEJ Siewert type I/II or EAC with cT2cN0 status at diagnosis undergoing multimodal treatment with FLOT or CROSS. Primary endpoint was overall survival (OS)

**Results:**

Between 2012 and 2023, 133 patients met the inclusion criteria, of whom 73 (54.9%) received CROSS and 60 (45.1%) underwent treatment with FLOT. In both groups, patients were mainly male (p = 0.08), older than 70 years (p = 0.24), and had American Society of Anesthesiologists (ASA) II classification (p = 0.45). Regarding surgical treatment, more patients underwent gastrectomy in the FLOT than in the CROSS cohort (23.3% versus 6.8%, p = 0.007). There were no differences regarding pT, pN, and pM category (p > 0.05). Median survival was not reached, while mean survival was 74.6 months (95% CI 60.5–88.7 months) for CROSS versus 100.8 months (95% CI 72.5–94.5 months, p = 0.028) for FLOT. The 3-year survival was 87% in the FLOT group versus 59% in the CROSS group. In multivariable analyses, FLOT was independent factor for survival (p < 0.001)

**Conclusions:**

For cT2cN0 staged EAC and GEJ type I /II patients FLOT chemotherapy showed a survival benefit and should be the preferred treatment in a multimodal approach.

**Supplementary Information:**

The online version contains supplementary material available at 10.1245/s10434-025-18311-8.

Whether perioperative chemotherapy according to the fluorouracil, leucovorin, oxaliplatin, and docetaxel (FLOT) protocol^1^ or neoadjuvant radiochemotherapy according to the Chemoradiotherapy for Oesophageal Cancer Followed by Surgery Study (CROSS) protocol^2^ should be preferred for adenocarcinoma (AC) of the esophagus (EAC) and gastroesophageal junction (GEJ) remained uncertain for decades. Despite multiple single- and multicenter retrospective analyses, a randomized trial showing a long-term survival benefit of one pretreatment in comparison with the other was debated until last year.^3–7^ However, the results of the randomized controlled ESOPEC study have been presented to the 2024 American Society of Clinical Oncology (ASCO).^8^ Here, FLOT showed a prolonged median overall survival in comparison with CROSS, with 66 months versus 37 months for the intention-to-treat population. In addition, the 5-year survival rate was 50.6% for the FLOT treatment in contrast to 38.7% for the CROSS protocol. The results of the study were just recently published and prompted an update of the European Society for Medical Oncology (ESMO) guidelines.^9,10^

In previous studies, more cardiopulmonary complications were shown for patients who received CROSS, while the number of patients with complete histopathological response was lower when treated according to FLOT.^5^ Patients with EGA or GEJ should in principal receive a multimodal approach, including radical resection for advanced tumor stages,^10^ however, this is still under debate for clinical stage 2 of the tumor, node, and metastasis (TNM) staging system.^10–13^ Therefore, countries such as the United Kingdom already pretreat stage cT2 EGA and GEJ^14^ to improve survival, as some patients with initial cT2 tumors showed postoperatively to be understaged with a histopathological stage higher than pT2.^15,16^ A recently published multicenter, European cohort study showed that a relevant number of initial stage cT2 patients are nowadays still understaged,^17^ possibly due to the fact that the staging modalities did not significantly improve during the last decade as indicated by a previous multicenter study from 2016.^18^ Considering the understaging, it must be concluded that patients who were understaged did not receive the optimal oncological therapy as compared with advanced tumors treated according to a multimodal approach with pretreatment according to either the CROSS or FLOT protocol.^1,2^

Given the potential long-term survival benefits of a multimodal approach for cT2 tumors,^17^ we performed a multicenter, European cohort analysis to investigate the hypothesis that one of these pretreatments might be superior for the specific subgroup of patients with stage cT2cN0 EGA and GEJ. This is particularly relevant as there was no distinct evaluation in earlier analysis as well as in the ESOPEC trial regarding the types of pretreatment used for stage cT2cN0 patients and their potential impact on survival outcomes.

## Patients and Methods

### Inclusion Criteria and Cohort Characteristics

Patients with primary adenocarcinoma of the esophagus and/or gastroesophageal junction Siewert types I and II with a clinical cT2 staging without positive lymph nodes (cN0) according to initial staging who had undergone neoadjuvant treatment according to the FLOT or CROSS regimens followed by elective surgery with curative intent between 2012 and 2023 (for the University of Cologne cohort; patients from 2016 onward) at one of the following ten European centers were included: Department of General, Visceral, Thoracic, and Transplant Surgery, University Hospital of Cologne; Department of Surgery, University Medical Center Utrecht; Department of Oesophagogastric Surgery, Churchill Hospital; Department of General, Visceral and Transplant Surgery, University Hospital of Heidelberg; Department of Surgery, St James’s Hospital Dublin; Department of General, Visceral, and Transplant Surgery, University Medical Center Mainz; Department of upper gastrointestinal diseases, Karolinska University Hospital; Department of Surgery, University Medical Center Amsterdam; Department of Surgery, Technical University of Munich; and Department of General, Visceral, Transplantation, Vascular, and Pediatric Surgery, University Hospital Wuerzburg.

**Fig. 1:**
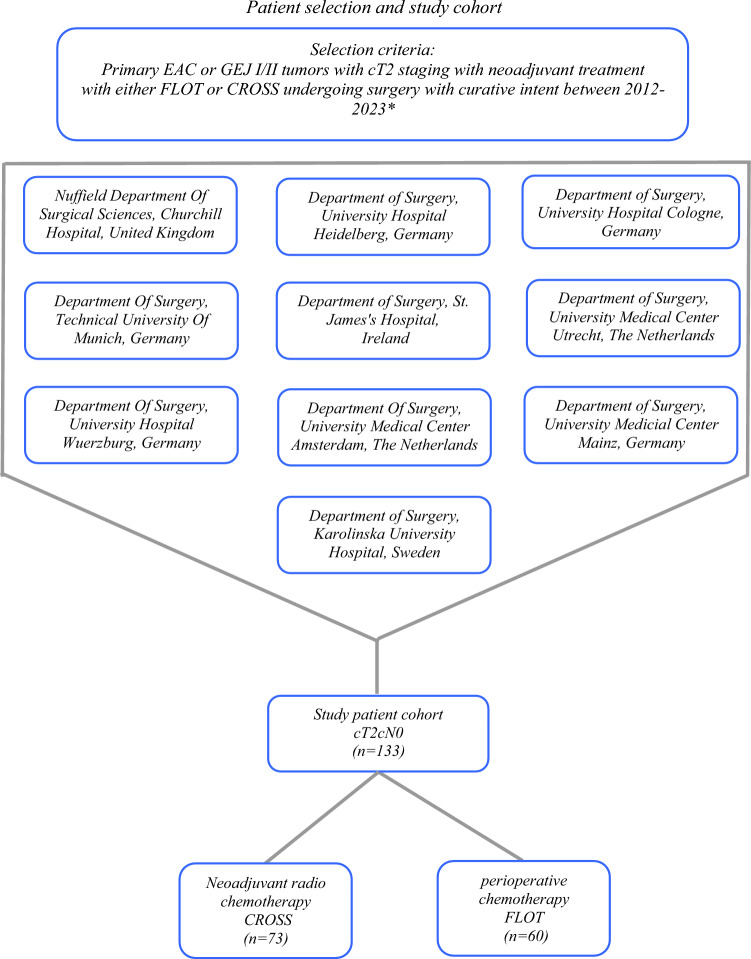
Overview of inclusion criteria and study cohort. Patients from University of Cologne were included from 2016 onwards

The clinicopathological factors and follow-up information of 133 patients fulfilling the previously mentioned inclusion criteria were collected from the prospective databases of the participating centers and were analyzed retrospectively (Fig. 1). All the participating institutes complied with their local ethics regulations.

### Staging

Clinical staging was performed using upper GI endoscopy with or without endoscopic ultrasound and biopsy for histological confirmation. The evaluation of distant metastases was performed with computed tomography (CT) of the chest and abdomen and/or positron-emission CT (PET-CT). If several staging modalities were used, cT2cN0 staging had to be confirmed in all.

### Pretreatment Characteristics

The localization of the tumor and the clinical stage were characterized according to the 8th edition of the Union International contre le cancer (UICC) staging system.^19^ The Physical Status Classification System of the American Society of Anesthesiologists (ASA)^20–22^ was used to characterize perioperative risks and preexisting comorbidities. In addition, patient characteristics of age, sex, body mass index (BMI), and staging modality were recorded.

### Surgical Techniques

Surgical technique, such as transhiatal extended gastrectomy or esophagectomy including open, hybrid, minimally invasive, or robot-assisted procedures (Supplementary Table 1), was dependent on tumor location. Perioperative complications were graded according to Clavien–Dindo classification.^23^

### Neoadjuvant Treatment

All patients (*n* = 133) received pretreatment before undergoing elective surgical resection. The treatments included perioperative chemotherapy according to the FLOT protocol (*n* = 60) or preoperative radiochemotherapy according to the CROSS protocol (*n* = 77). The FLOT protocol consists of a chemotherapy with four preoperative 2-week cycles of docetaxel, oxaliplatin, leucovorin, and fluorouracil,^24^ whereas the CROSS protocol includes five cycles of neoadjuvant chemotherapy with carboplatin and paclitaxel with concurrent radiotherapy (41.4 Gy, given in 23 fractions of 1.8 Gy).^25^

### Histopathology and Postoperative Staging

The resected histopathological specimens were analyzed by the pathology departments of the participating centers. Histopathological staging was performed according to the 8th edition of the UICC classification with inclusion of the extent of the primary tumor, involvement of the regional lymph nodes, and confirmation of distant metastases ((y)pTNM), as well as radicality and tumor regression grade.

### Follow-Up

Follow-up was completed in August 2023. Complete follow-up information was available for all the patients. At the last follow-up, 30 of the 133 patients (22.6%) had died.

The primary endpoint of this study was median overall survival between neoadjuvant treatment with chemotherapy according to the FLOT protocol or radiochemotherapy according to the CROSS protocol. Secondary outcomes included disease-free survival and radicality.

### Statistical Analysis

Overall survival (OS) was calculated from the time of surgery until death or last follow-up. Disease-free survival (DFS) was calculated from the time of surgery until recurrence or death or last follow-up. Survival curves were plotted using the Kaplan–Meier method. To compare categorical variables, the *χ*^2^ and Fisher’s exact tests were used. The Mann–Whitney *U* test was applied to compare independent sample sizes. Propensity score matching (PSM) was performed for patient characteristics including age, ASA classification, body mass index, type of resection, and sex. One-to-one matching without replacement was performed using a 0.1 caliper width and the resulting score-matched pairs were used in subsequent analyses as indicated. Prognostic factors were evaluated by univariate and multivariate analyses using the Cox regression model. All tests were two-sided, and a *p*-value < 0.05 was considered statistically significant. Analyses were performed using SPSS version 29.0 (SPSS Inc., Chicago, Illinois, USA).

## Results

### Pretreatment Patient and Tumor Characteristics

Regarding patient characteristics, the majority in the CROSS- as well as in the FLOT-treated cohort were male (91.8% versus 81.7%, *p* = 0.08), older than 70 years at time of surgery (58.9% versus 48.3%, *p* = 0.24, and with a BMI between 25 kg/m^2^ and 29 kg/m^2^ (47.2% versus 45.6%, *p* = 0.19). Preoperative morbidity measured by ASA classification was equally distributed over the cohorts (*p* = 0.4). Preoperative tumor characteristics were similar, as only clinically staged cT2cN0 tumors were included, while the tumor localization was different between the two treatment arms. The CROSS protocol was more frequently used in Siewert type I tumors (61.6% versus 36.7%, *p* = 0.008; see Table 1).

**Table 1:** Patient characteristics pretreatment

Clinicopathological Factor	CROSS (*n* = 73)	FLOT (*n* = 60)	Total (*n* = 133)	*P*-value
Age at diagnosis (years)*				0.44
	66 (57–74)	65 (57–70)	66 (57–72)	
Age				0.24
≤ 45	2 (2.7%)	5 (8.3.%)	7 (5.3%)	
46–69	28 (38.4%)	26 (43.3%)	54 (40.6%)	
> 70	43 (58.9%)	29 (48.3%)	72 (54.1%)	
Sex				0.08
Female	6 (8.2%)	11 (18.3%)	17 (12.8%)	
Male	67 (91.8%)	49 (81.7%)	116 (87.2%)	
ASA classification**				0.45
ASA I	12 (16.4%)	8 (13.3%)	20 (15.0%)	
ASA II	37 (50.7%)	26 (43.3%)	63 (47.4%)	
ASA III	23 (31.5%)	26 (43.3%)	49 (36.8%)	
ASA IV	0 (0.0%)	0 (0.0%)	0 (0%)	
BMI**				0.19
< 25	23 (31.9%)	12 (21.1%)	35 (27.1%)	
25–29	34 (47.2%)	26 (45.6%)	60 (46.5%)	
≥ 30	15 (20.8%)	19 (33.3%)	34 (26.4%)	
Tumor localisation				**0.008**
Esophageus	9 (12.3%)	18 (30.0%)	27 (18.4%)	
Siewert type I	45 (61.6%)	22 (36.7%)	67 (48.0%)	
Siewert type II	19 (26.0%)	20 (33.3%)	39 (33.5%)	
cT-category				
cT2	73 (100%)	60 (100%)	133 (100.0%)	
cN-category				
cN0	73 (100%)	60 (100%)	133 (100.0%)	
cM-category				0.89
cM0	72 (98.6%)	59 (98.3%)	131 (98.6%)	
cMx	1 (1.4%)	1 (1.7%)	2 (1.4%)	

*median (range); **Data was not available for all patients; values in bold print indicate a significance-level of *p* ≤ 0.05

### Surgery and Perioperative Morbidity

For all patients the predominantly performed surgical approach was esophagectomy (85.7*%)*, while more patients underwent a transhiatal extended gastrectomy in the FLOT group than in the CROSS group (23.3% versus 6.8%, *p* = 0.007). Comparing both cohorts, there were no relevant differences regarding postoperative morbidity evaluated with the Clavien–Dindo classification (*p* = 0.43). The median stay in the intensive care unit as well as the median hospital stay in total was comparable between the FLOT and the CROSS groups (*p* = 0.22 versus *p* = 0.16; see Table 2).

**Table 2:** Postoperative outcomes and tumor histopathology of cT2cN0 patients

Clinicopathological Factor	CROSS (*n* = 73)	FLOT (*n* = 60)	total (*n* = 133)	*P*-value
Surgery type				**0.007**
Esophagectomy	68 (93.2%)	46 (76.7%)	114 (85.7%)	
Gastrectomy	5 (6.8%)	14 (23.3%)	19 (14.3%)	
Morbidity**				0.43
Clavien-Dindo 0	22 (30.1%)	25 (41.7%)	47 (35.3%)	
Clavien-Dindo I+II	21 (28.8%)	18 (30.0%)	39 (29.3%)	
Clavien-Dindo IIIa	11 (15.1%)	6 (10.0%)	17 (12.8%)	
Clavien-Dindo ≥ IIIb	19 (26.0%)	11 (18.3%)	30 (22.6%)	
ICU stay (days)	2 (1-3)	1 (1-2)	2 (1-3)	0.22
hospital stay (days)	13 (9-22)	11 (8-15)	11 (9-19)	0.16
pT-category				0.53
pT0	7 (9.6%)	8 (13.3%)	15 (11.3%)	
pT1	26 (35.6%)	19 (31.7%)	45 (33.8%)	
pT2	15 (20.5%)	10 (16.7%)	25 (18.8%)	
pT3	24 (32.9%)	21 (35.0%)	45 (33.8%)	
pT4	0 (0.0%)	2 (3.3%)	2 (1.5%)	
pTx	1 (1.4%)	0 (0%)	1 (0.8 %)	
pN-category				0.10
pN0	49 (67.1%)	35 (58.3%)	84 (63.2%)	
pN1	19 (26.0%)	12 (20.0%)	31 (23.3%)	
pN2	3 (4.1%)	8 (13.3%)	11 (8.3%)	
pN3	2 (2.7%)	5 (8.3%)	7 (5.3%)	
Number of tumor positive lymph nodes	0 (0-1)	0 (0-2)	0 (0-1)	0.39
Number of resected lymph nodes	31 (20-37)	35 (23-43)	30 (21-40)	0.19
pM-category				0.89
pM0	72(98.6%)	59 (98.3%)	131 (98.5%)	
pM1	1 (1.4%)	1 (1.7%)	2 (1.5%)	
R resection rate				0.51
R0	70 (95.9%)	56 (93.3%)	126 (94.7%)	
R1	3 (4.1%)	4 (6.7%)	7 (5.3%)	
Tumor regression grade*				0.16
Complete	7 (11.1%)	6 (11.1%)	13 (11.1%)	
Major	9 (14.3%)	14 (25.9%)	23 (19.7%)	
Partial	34 (54.0%)	14 (25.9%)	48 (41.0%)	
Minor	13 (20.6%)	20 (27.1%)	33 (28.2%)	

*median (range); **Data was not available for all patients; values in bold print indicate a significance-level of *p* ≤ 0.05

### Histopathological Outcomes

Despite clinical staging being cT2cN0 for all included patients, the main pathological T stage was pT1 and pT3 with 33.8% each (*p* = 0.53), and only 18.8% of all patients had pT2 stage, with no relevant differences between CROSS- or FLOT-treated patients (*p* = 0.53).

Furthermore, there was a tendency of more tumor-positive lymph nodes in the FLOT-treated group with pN2 category of 13.3% versus 4.1% in comparison with the CROSS-treated patients, without being significant. Around 63.2% of all patients included had no lymph node metastases, which suggests that at least 36.8% of all patients were initially understaged regarding lymph node involvement. The median number of resected and tumor-positive lymph nodes was similar in both cohorts (*p* = 0.39 versus *p* = 0.19; see Table 2).

There were no differences in the pM category (*p* = 0.89) and the radical resection rate (*p* = 0.51) between the FLOT and the CROSS groups. There were no relevant distinctions when comparing tumor histopathological regression grades in both groups (*p* = 0.16; see Table 2).

### Long-Term Oncologic Outcome and Survival

**Fig. 2:**
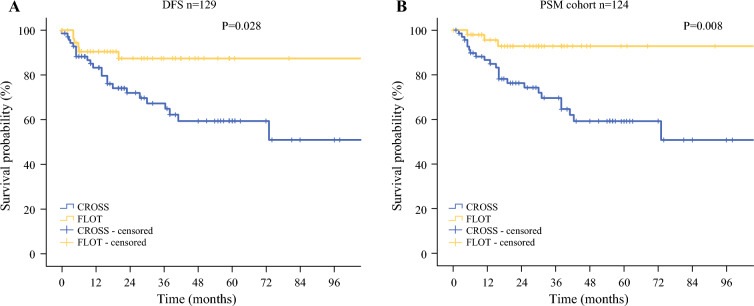
Kaplan-Maier plots for survival of patients with cT2cN0 staged EAC or GEJ type I/II undergoing either a pretreatment with FLOT or CROSS. (A) Overall survival of all patients undergoing FLOT or CROSS (n=60/73;* p* = 0.028), (B) Overall survival of all patients pretreated with FLOT or CROSS after PSM (n=51/73; p=0.008).

Median follow-up was 31 months for all patients. Interestingly, the patients undergoing pretreatment with perioperative chemotherapy according to the FLOT protocol showed longer overall survival than the patients undergoing pretreatment with radiochemotherapy according to the CROSS protocol, while both cohorts did not reach the median overall survival (Fig. 2A, p = 0.028). The mean survival was 74.6 months (95% CI 60.5–88.7 months) for patients undergoing CROSS treatment versus 100.8 months (95% CI 72.5–94.5 months) for patients receiving FLOT treatment. The 3-year survival was 59% in the CROSS group in contrast to 87% in the FLOT group. The 5-year survival rate was comparable, with 50.8% in the CROSS-treated cohort versus 87% in the FLOT-treated cohort.

Furthermore, we performed propensity score matching (PSM) to balance the preoperative characteristics and the surgical treatment between both treatment groups (Supplementary Table 2), where FLOT-treated patients still had a survival benefit in contrast to those undergoing CROSS treatment (*p* = 0.008, Supplementary Fig. 1B).

To account for the differences in surgical approach between the two pretreatment groups of the full cohort, we excluded all patients undergoing gastrectomy und reanalyzed overall survival, which remained significantly higher for the patients who received FLOT in comparison with CROSS as neoadjuvant treatment (*p* = 0.019, Supplementary Fig. 1A). In addition, FLOT treatment was also associated with survival benefit in respect to disease-free survival in contrast to CROSS treatment (*p* = 0.028, Supplementary Fig. 1B).

### Multivariable Analysis

In multivariable analysis, pretreatment with FLOT was an independent prognostic factor for survival, together with pT, pN, and pM category (*p* < 0.001, Table 3A). Perioperative chemotherapy with FLOT remained an independent prognostic factor after PSM of the study cohort (*p* < 0.001, Table 3B), indicating that pretreatment, independent of surgical approach, has an influence on patient survival.

**Table 3:** (A) cT2 cNo cohort (*n* = 133), (B) PSM cT2 cNo cohort (*n* = 124)

Variables			Multivariable analysis inclusion		
	B	HR	95% CI		*P*-value
(A) cT2 cNo cohort (*n* = 133)					
CROSS/FLOT	−2.09	0.12	0.04	0.37	<0.001
Pathological T stage	0.40	1.49	1.03	2.16	0.03
Pathological N stage	−0.90	2.45	1.59	3.80	<0.001
Pathological M stage	−3.45	0.03	0.01	0.18	<0.001
(B)PSM cT2 cNo cohort (*n* = 124)					
CROSS/FLOT	−2.75	0.06	0.02	0.26	<0.001
Pathological T stage	0.44	1.55	1.04	2.30	0.03
Pathological N stage	1.09	2.98	1.86	4.78	<0.001
Pathological M stage	−3.95	0.02	0.01	0.13	<0.001

B: Coefficient, HR: Hazard Ratio; CI: Confidence Intervall

## Discussion

In this study, perioperative FLOT therapy showed a survival benefit in comparison with neoadjuvant radiochemotherapy following the CROSS protocol for the stage cT2cN0 patient subgroup with adenocarcinoma of the esophagus as well as of the esophagogastric junction Siewert types I and II. Despite being a small cohort, this study is the first to show relevant survival differences depending on the pretreatment applied for this group of stage cT2N0 patients. Even nowadays, it is extremely difficult to evaluate cT2cN0 patients, as they remain a minority among patients with EAC and GEJ, especially in non-high-volume centers.^26^

Only last year were we able to retrospectively show that cT2cN0 patients undergoing surgical resection in different European high-volume centers benefit from pretreatment in terms of survival. There still have not been any relevant randomized controlled trials undertaken due to the low number of patients.^17^ Besides the low number of cT2cN0 patients, another main difficulty in characterizing this subgroup is inadequate staging despite the use of various staging modalities.^26,27^

Nonetheless, previous studies did not analyze potential differences that the type of pretreatment may have on cT2cN0 patients,^12,13,15,17,18^ as previous studies showed no distinction between treatments such as FLOT and CROSS.^3–5^ The randomized controlled trial ESOPEC was able to show a survival benefit for FLOT in comparison with CROSS, while not analyzing subgroups such as cT2cN0 patients separately.^9^ Randomized controlled trials for multimodal treatments of stage cT2cN0 cohorts are still lacking^17,18^ and may even never be undertaken.

Interestingly, in this cohort, histopathological lymph nodal involvement was more frequent for patients undergoing FLOT than CROSS, while not reaching significance. These results are comparable to the cohort of the ESOPEC study, where more lymph node positive patients were in the FLOT than the CROSS group (58% versus 46%).^9^

FLOT has potentially more systemic effect than CROSS neoadjuvant radiochemotherapy and therefore has a potential higher impact on long-term survival. It has been shown that patients with initially staged lymph node disease have a similar survival prognosis as the ones that had none at all, if they responded to chemotherapy with full regression of their lymph node metastasis.^28^ Considering that at least 30% of this study cohort were understaged as cN0, it could be understandable that FLOT has this observed impact on patient survival, as those who responded to pretreatment would have similar survival chances to those who had no lymph node involvement prior to neoadjuvant treatment.

Therefore, the systemic effect may be more relevant for long-term survival than local regression of the primary tumor, as even compared with full responders of the primary tumor, the patients with persistent histopathological lymph nodal involvement had a significant lower survival than the ones without, thereby showing the relevance of lymph node response to neoadjuvant treatment.^29^ Sadly, there are still a relevant number of patients who do not respond to either of those two treatments,^2,27^ as shown in this study where the number of minor responders was more than 20% in both cohorts.

The question of whether perioperative application of FLOT in comparison with the only neoadjuvant applied CROSS is relevant to improving patient survival remains undecided, especially as the SPACE FLOT study showed that the application of adjuvant FLOT did not improve survival for patients with relevant histopathological response to neoadjuvant FLOT.^30^

To address potential bias of surgical treatment, as the number of gastrectomies was higher in the FLOT group of this study, subgroup analysis and PSM were performed, but a clear survival benefit for FLOT in comparison with CROSS was still observed.

Nonetheless, this was a small and retrospective study, which limits the reliable conclusions that can be made. However, the results of this study seem to have high potential value in the improvement of treatment of cT2cN0 patients and their long-term survival, as the 3-year survival rate was 87% for stage cT2cN0 patients undergoing FLOT treatment compared with 57.4% of the FLOT-treated patients in the ESOPEC trial,^9^ showing that even with a relevant number of understaged patients, FLOT considerably improved survival rates of this specific subgroup. Comparing the 3-year survival rates between our study and the ESOPEC trial for CROSS pretreatment, CROSS showed similar 3-year survival rates, with 59% in the cT2cN0 cohort versus 50.7% of the ESOPEC study cohort, underlining that understaging of cT2cN0 patients, especially regarding lymph node involvement, could potentially be reduced through the application of CROSS than FLOT neoadjuvantly.^28^ Therefore, our study shows the relevance of FLOT even in early tumor stages, as underestimated lymph node disease remains a fundamental disadvantage of patients with EAC and GEJ types I and II. The application of artificial intelligence may potentially be used to improve clinical staging and support clinicians in the allocation process regarding oncological treatment in the years to come.^31^

## Conclusions

Given that more than a third of patients with EAC and GEJ type I and II with clinical cT2cN0 stage are understaged and may benefit from multimodal treatment, this study highlights the potential advantage of perioperative chemotherapy according to the FLOT protocol over radiochemotherapy according to the CROSS protocol for improving long-term survival.

## Supplementary Information

Below is the link to the electronic supplementary material.

Supplementary file1 (DOCX 430 kb)

## Data Availability

Datasets generated and analyzed for this work are not publicly available, but are accessible upon request.
