# Supplementary material for: Comparison of Pretreatment Strategies for cT2cN0 Staged Adenocarcinoma of the Esophagus and the Gastroesophageal Junction: A European High-Volume Center Cohort Analysis
Source: Ann Surg Oncol. 2025 Oct 13;32(13):10128–36. doi: 10.1245/s10434-025-18311-8 (PMC12589201; doi:10.1245/s10434-025-18311-8)
Supplement: Supplementary file 1 — Supplementary file1 (DOCX 430 kb) [file 10434_2025_18311_MOESM1_ESM.docx]

*Supplementary Figure 1*

Kaplan-Maier plots for survival of patients with cT2cN0 staged EAC or GEJ type I/II undergoing either a pretreatment with FLOT or CROSS. (A) Overall survival of all patients undergoing esophagectomy pretreated either with FLOT or CROSS (n=48/68; p=0.019). (B) Disease-free survival of all patients undergoing a pretreatment with FLOT or CROSS (n=57/72; p=0.028).


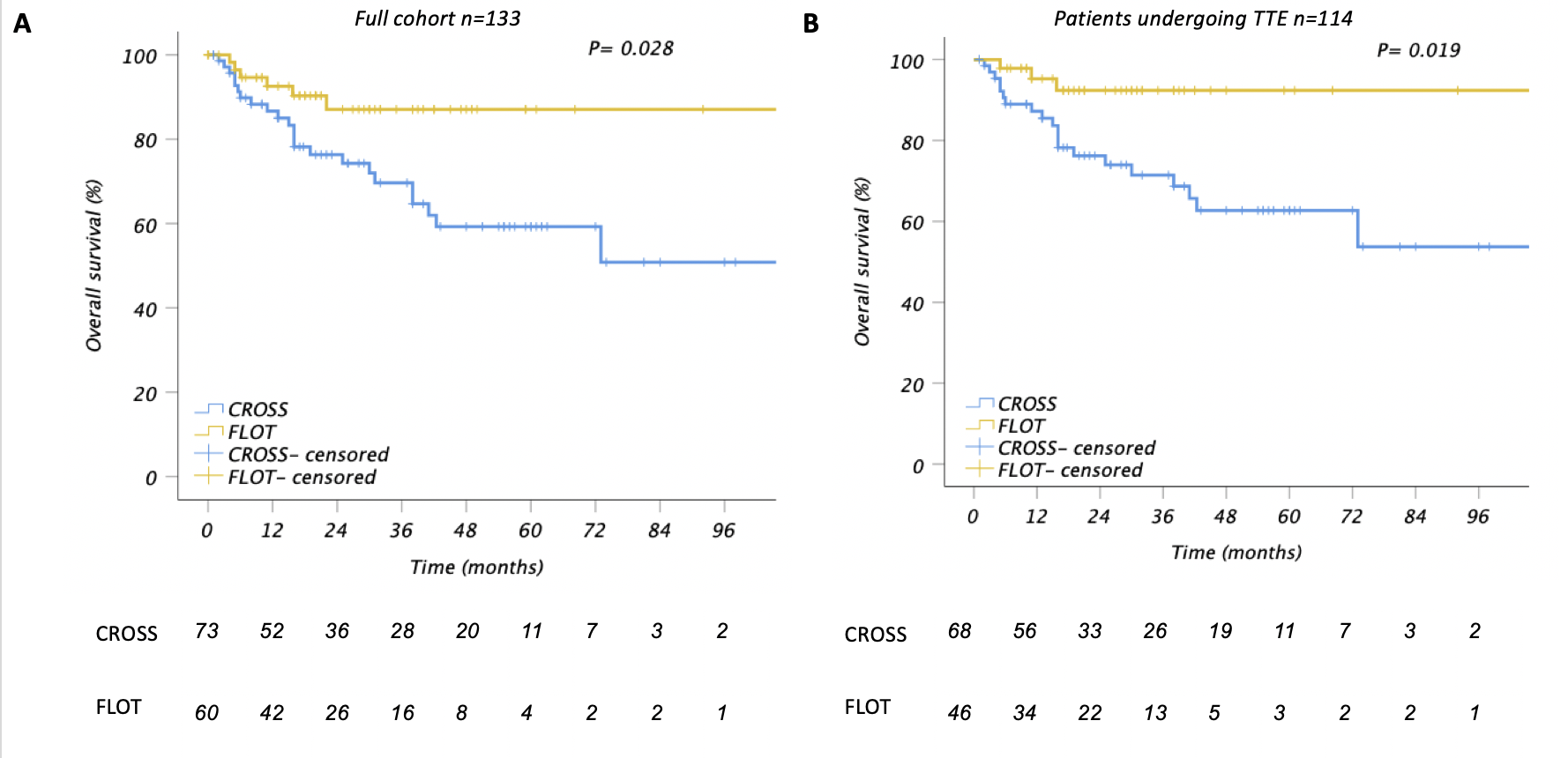


**Suppl. Table 1. Surgical technique**

| **Clinicopathological Factor** | **CROSS**  **(n=73)** | **FLOT**  **(n=60)** | **total**  **(n=133)** | **P-value** |
| --- | --- | --- | --- | --- |
| **Surgical technique** |  | | | 0.15 |
| open | 24 (32.9%) | 30 (50.0%) | 54 (40.6%) |  |
| hybrid | 8 (11.0%) | 7 (11.7%) | 15 (11.3%) |  |
| laparoscopic | 26 (35.6%) | 12 (20.0%) | 38 (28.6%) |  |
| robotic assisted | 15 (20.5%) | 11 (18.3%) | 26 (19.5%) |  |

values in bold print indicate a significance-level of p≤0.05

**Suppl. Table 2. PSM cohort (n=124)**

| **Clinicopathological Factor** | **CROSS**  **(n=73)** | **FLOT**  **(n=51)** | **total**  **(n=124)** | **P-value** |
| --- | --- | --- | --- | --- |
| **Age** |  | | | 0.26 |
| ≤45 | 2 (2.7%) | 4 (7.8%) | 6 (4.8%) |  |
| 46-69 | 28 (38.4%) | 23 (45.1%) | 51 (41.1%) |  |
| >70 | 43 (58.9%) | 24 (47.1%) | 67 (54.0%) |  |
| **Sex** |  | | | 0.20 |
| Female | 6 (8.2%) | 8 (15.7%) | 14 (11.3%) |  |
| Male | 67 (91.8%) | 43 (84.3%) | 110 (88.7%) |  |
| **ASA classification**** |  | | | 0.51 |
| ASA I | 12 (16.4%) | 7 (13.7%) | 19 (15.3%) |  |
| ASA II | 37 (50.7%) | 22 (43.1%) | 59 (47.6%) |  |
| ASA III | 23 (31.5%) | 22 (43.1%) | 45 (36.3%) |  |
| ASA IV | 0 (0.0%) | 0 (0.0%) | 0 (0%) |  |
| **BMI**** |  |  |  | 0.12 |
| <25 | 23 (31.9%) | 9 (18.8%) | 32 (26.7%) |  |
| 25-29 | 34 (47.2%) | 22 (45.8%) | 56 (46.7%) |  |
| ≥30 | 15 (20.8%) | 17 (35.4%) | 32 (26.7%) |  |
| **OP type** |  | | | 0.55 |
| esophagectomy | 68 (93.2%) | 46 (90.2%) | 114 (91.4%) |  |
| gastrectomy | 5 (6.8%) | 5 (9.8%) | 10 (8.1%) |  |

**Data was not available for all patients; values in bold print indicate a significance-level of p≤0.05

**Suppl. Table 3. Localisation of Recurrences**

| **Clinicopathological Factor** | **CROSS**  **(n=22)** | **FLOT**  **(n=12)** | **total**  **(n=34)** | **P-value** |
| --- | --- | --- | --- | --- |
| **Localisation** |  | | | 0.39 |
| local | 4 (18.2%) | 3 (25%) | 7 (20.5%) |  |
| lymph nodal | 2 (9.1%) | 2 (16.7%) | 4 (11.8%) |  |
| liver | 0 (0%) | 1 (8.3%) | 1 (2.9%) |  |
| lung | 6 (27.3%) | 0 (%) | 6 (17.7%) |  |
| other | 7 (31.8%) | 4 (33.3%) | 11 (32.4%) |  |
| multiple sites | 3 (13.6%) | 2 (16.7%) | 5 (14.7%) |  |

values in bold print indicate a significance-level of p≤0.05
